# The Clinical and Prognostic Impact of the Choice of Surgical Approach to Fourth Ventricular Tumors in a Single-Center, Single-Surgeon Cohort of 92 Consecutive Pediatric Patients

**DOI:** 10.3389/fonc.2022.821738

**Published:** 2022-02-24

**Authors:** Nicola Onorini, Pietro Spennato, Valentina Orlando, Fabio Savoia, Camilla Calì, Carmela Russo, Lucia De Martino, Maria Serena de Santi, Giuseppe Mirone, Claudio Ruggiero, Lucia Quaglietta, Giuseppe Cinalli

**Affiliations:** ^1^ Department of Pediatric Neurosciences, Pediatric Neurosurgery Unit, Santobono-Pausilipon Children’s Hospital, Naples, Italy; ^2^ Division of Neurosurgery, Department of Neurosciences, Reproductive and Odontostomatological Sciences, Università degli Studi di Napoli Federico II, Naples, Italy; ^3^ Evaluative Epidemiology-Childhood Cancer Registry of Campania, Santobono-Pausilipon Children’s Hospital, Naples, Italy; ^4^ Department of Pediatric Neurosciences, Pediatric Neuroradiology Unit, Santobono-Pausilipon Children’s Hospital, Naples, Italy; ^5^ Department of Pediatric Neurosciences, Pediatric Neuro-Oncology Unit, Santobono-Pausilipon Children’s Hospital, Naples, Italy

**Keywords:** fourth ventricle, telovelar, transvermian, cerebellar mutism, children

## Abstract

**Objective:**

A single-institution cohort of 92 consecutive pediatric patients harboring tumors involving the fourth ventricle, surgically treated *via* the telovelar or transvermian approach, was retrospectively reviewed in order to analyze the impact of surgical route on surgery-related outcomes and cumulative survival.

**Methods:**

Clinical, radiological, surgical, and pathology details were retrospectively analyzed. We selected n = 6 surgery-related clinical and radiological outcomes: transient and permanent neurological deficits, duration of assisted ventilation, postoperative new onset medical events, postoperative cerebellar mutism, and extent of resection. We built univariate and multivariate logistic models to analyze the significance of relationships between the surgical routes and the outcomes. Cumulative survival (CS) was estimated by the cohort approach.

**Results:**

There were 53 girls and 39 boys (mean age, 83 months). Telovelar approach was performed in 51 cases and transvermian approach in 41 cases. Early postoperative MRI studies showed complete removal in 57 cases (62%) and measurable residual tumor in 35 cases (38%). The average tumor residual volume was 1,316 cm^3^ (range, 0.016–4.231 cm^3^; median value, 0.9875 cm^3^). Residual disease was more often detected on immediate postop MRI after telovelar approach, but the difference was not significant. Cerebellar mutism was observed in 10 cases (11%). No significant difference in the onset of cerebellar mutism was detected between telovelar and transvermian approach. The choice of surgical approach did not significantly modify any other postoperative outcome and 1-/3-year CS of high-grade surgically treated tumors.

**Conclusions:**

With the limitation of a single-center, single-surgeon retrospective series, our findings offer significant data to reconsider the real impact of the choice of the surgical route to the fourth ventricle on the incidence of cerebellar mutism and surgery-related morbidity. This seems to be in line with some recent reports in the literature. Surgical approach to the fourth ventricle should be individualized according to the location of the tumor, degree of vermian infiltration, and lateral and upward extension. Telovelar and transvermian approaches should not be considered alternative but complementary. Pediatric neurosurgeons should fully master both approaches and choose the one that they consider the best for the patient based on a thorough and careful evaluation of pre-operative imaging.

## Introduction

The surgical strategy to access the fourth ventricle has evolved over time in order to minimize the surgical invasiveness and maximize the degree of surgical resection (1–5). Along this line, the telovelar approach (6–11) was introduced to avoid the anatomical damage of the classic transvermian route (3, 4, 8) and the potential consequences in terms of postoperative cerebellar mutism (12, 13).

The aim of our study was to analyze the impact of the choice of the surgical route to the fourth ventricle on the incidence of cerebellar mutism (CM) and surgery-related morbidity in children.

## Materials and Methods

### Patient Population: Criteria of Inclusion/Exclusion

From January 2007 to June 2018, 215 patients below 18 years of age were operated for neoplasms of the posterior fossa at the Department of Pediatric Neurosurgery of Santobono-Pausilipon Children’s Hospital of Naples. The patient was considered eligible for this study if 1) there is primary diagnosis without previous surgeries, 2) the tumor was located into the fourth ventricle, 3) it invaded the fourth ventricle from adjacent anatomical structure, or 4) its removal required surgical approach to the fourth ventricle.

Exclusion criteria were the following: 1) relapsed or progressing cases first treated before January 2007, 2) cerebellar hemispheric tumors, 3) brainstem tumors without predominant (>50%) exophytic component into the fourth ventricle, 4) tumors of the cerebellopontine angle without fourth ventricular involvement, 5) pineal tumor, and 6) purely aqueductal tumors.

The retrospective analysis of our study covers a period from January 2007 to June 2018. The Senior author started using the telovelar approach from 2001. The tumors of the fourth ventricle treated from 2001 to 2006 were excluded from our analysis in order to avoid the bias of the possible conversion from the telovelar to the transvermian approach.

**Figure 1:**
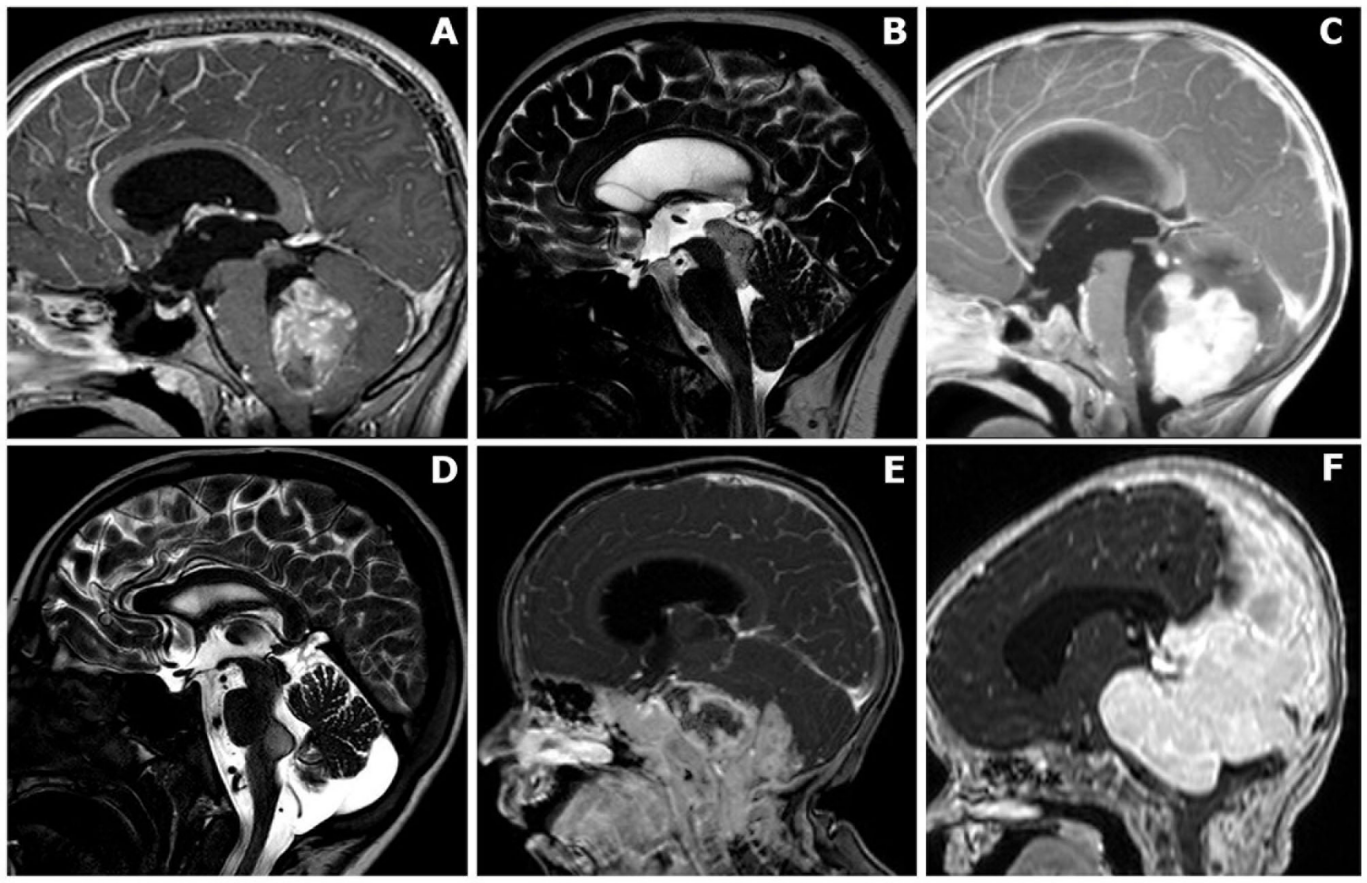
Anatomical classification of posterior fossa tumors requiring surgical access to the fourth ventricle**. (A)** Mainly/purely intraventricular, without evident brainstem infiltration or extensive vermian infiltration. **(B)** Midbrain/intra-aqueductal tumor with significant bulging in the upper part of the fourth ventricle. **(C)** Cerebellar/vermian tumor with extensive parenchymal/vermian infiltration and secondary bulging into the fourth ventricle. **(D)** Cerebellopontine angle tumors extending into the fourth ventricle through the Luschka foramen/foramina. **(E)** Brainstem tumors with dorsally exophytic fourth ventricular component. **(F)** Giant tumors with extensive posterior fossa involvement, including a significant fourth ventricle component..

The selected cases were classified in the following six main groups: (A) intraventricular, (B) mesencephalo-aqueductal tumors, (C) cerebellar/vermian tumors, (D) cerebellopontine angle tumors extending to the fourth ventricle, (E) brainstem tumors with exophytic fourth ventricular component, and (F) large tumors with extensive posterior fossa involvement, including the fourth ventricle (**Figure 1**).

### Clinical and Neuroimaging Data

Medical records, neuroimaging studies, and operative and pathological reports were retrospectively analyzed.

Clinical and neurological status before surgery and new transient (<1 year after surgery) or permanent (>1 year after surgery) postoperative neurological deficits and cerebrospinal fluid (CSF)-related complications were identified. Postoperative infections, new onset medical conditions, days of mechanical ventilation, and intensive care unit length of stay were also analyzed.

Before 2016, we identified CM in cases of muteness with delayed onset and limited duration, following posterior fossa surgery, usually presenting with other neurological sings/symptoms: emotional lability, hypotonia, ataxia, long tract sings, and cranial nerve palsy. Since 2016, the Iceland Delphi Group diagnostic criteria (14) for CM have been adopted in our Institution.

All patients with postoperative CM underwent early postoperative neuropsychological assessment (<72 h) exploring: attention, memory, executive functions, processing speed, and cognitive efficiency (although in absence of a standardized institutional protocol) and also full preoperative and late postoperative (1, 3, and 12 months) complete neuropsychological assessment.

All patients were studied using brain CT scan and/or brain and spinal cord contrast-enhanced MRI scan on a 1.5-T machine. MRI sequences used for anatomical classification were T1w without and with injection, T2w turbo spin-echo, T2w fast spin-echo, DRIVE, and balanced fast-field echo (B-FFE). These sequences allowed to classify uni- or bilateral involvement of the foramina of Luschka, caudal extension through the foramen of Magendie, vermian and brainstem infiltration, and cerebellar tonsillar herniation below the foramen magnum.

All patients received postoperative MRI within 24 h to assess the following: extent of resection (EOR%), residual tumor volume (cm^3^) and location, and postoperative complications. Flow void sign through the endoscopic third ventriculostomy (ETV) was sought on T2-w sagittal cuts.

Tumor preoperative volume (cm^3^) and tumor residual volume (cm^3^) were calculated for each patient independently by two authors (NO and VO) using 3D volumetric sequences in Horos TM 3.3.5 (GNU General Public License, version 3.0) on the basis of axial sections on 3D T1-w FFE contrast-enhanced images. In case of computational disagreement, an arbitrary difference of 5% of the largest calculation was set as the limit for revision by a third author (CR). Preoperative tumor volumes were split into three homogeneous groups using two tertiles.

### Surgery

Every attempt was done not to open the posterior fossa in the presence of untreated hydrocephalus. Endoscopic third ventriculostomy (ETV), external ventricular drain (EVD), or ventriculoperitoneal (VP) shunt were performed, if necessary, at presentation by the neurosurgeon on call at the time of admission in agreement with senior author indications, depending on clinicoradiological features (age, metastases, clinical conditions, anatomy of third ventricle floor, and available surgical theater).

All patients were operated in the prone position through a median suboccipital craniotomy. The senior author decided the surgical approach on preoperative neuroimaging without randomization.

Intraoperative macroscopic evidence of vermian and/or brainstem infiltration and extension of cervical laminectomy/laminotomy, dural opening, and closing of posterior fossa dural defect were noted. Anesthesia records provided information on clinical parameters during surgery.

Surgical microscope was used in all cases, and all procedures were recorded (Zeiss^®^ OPMI^®^/NC4 microscope; Zeiss^®^ OPMI^®^/Pentero^®^ 800 microscope).

Since 2009, intraoperative neuronavigation system was routinely used (Medtronic^®^ StealthStation Treon Plus^®^ Surgical Navigation System; Medtronic^®^ StealthStation S7^®^ and S8^®^ Surgical Navigation System). The CUSA^®^ Excel^®^ ultrasonic aspiration system was used in all cases, changing the irrigation, aspiration, amplitude, and tissue select modes in relation to the specific situation. Self-retaining flexible retractors are adopted in all procedures.

Neurophysiological intraoperative monitoring (IOM) was used in case of dorsal exophytic brainstem tumor, brainstem infiltration, fourth ventricle tumor with cerebellopontine angle (CPA) involvement, and tumor extending through the foramen magnum.

### Follow-Up and Adjuvant Therapy

All follow-up MRI studies were retrospectively reviewed to assess the presence and timing of recurrence or regrowth of residual tumor. The timing of follow-up MRI was adapted according to pathological results and intercurrent modifications of neurological status. We considered as the most recent follow-up the date of the last MRI study and consequent neurosurgical/oncological examination.

Patients enrolled in this study were all evaluated by a multidisciplinary team and treated according to national guidelines. Treatment protocols of the Italian Society of Pediatric Oncology were followed for adjuvant treatment in case of medulloblastoma, ependymoma, and AT-RT. Standard treatment options for childhood low-grade gliomas included follow-up and surgery in case of residual tumor or recurrence; radiotherapy was rarely indicated and only in case of failure of surgery to achieve complete resection or if surgery was considered too dangerous for neurological function.

### Statistical Analysis

Six post-surgical outcomes were analyzed: transient and permanent neurological deficits (respectively: <1 and >1 year after surgery), duration of assisted ventilation (two groups with 48 h cut-off), postoperative new onset medical events (yes–no), postoperative CM (yes–no), and extent of resection (three groups: no residual disease, residual volume <1.5 or > 1.5 cm^3^).

We built univariate and multivariate logistic models for every outcome. We calculated odds ratios (OR) and 95% confidence interval (95% CI), and we analyzed the significance of relationships between the outcomes and the covariate variables (p < 0.05 was considered significant). In logistic regression, the maximum likelihood estimation suffered from small-sample bias, so in our analysis, we choose to use the penalized maximum likelihood estimation proposed by Firth: always in multivariate analysis and in the univariate analysis when one of the number of events resulted equal to zero. The postoperative tumor residual volume is an ordinal outcome, so the ordered logistic model was applied to estimate its relationship with the set of covariates (both in univariate and multivariate analysis).

Cumulative survival (CS) was estimated by the cohort approach. One- and 3-year cumulative survival was analyzed in relation to histological grading (WHO 1–2 vs. WHO 3–4), histological subtype (medulloblastoma, pilocytic astrocytoma, and ependymomas), and surgical approach (telovelar approach–transvermian approach for high-grade tumors) and was calculated using the Kaplan–Meier method. The confidence intervals (95% CI) of the survivor functions were obtained using Greenwood’s formula. The survival distributions were compared using the log-rank test (p < 0.05 was considered significant). For the surgical approach, the power of predicting factors (surgical approach and postoperative residual tumor volume) was evaluated in a Cox proportional hazard model.

The analyses were performed using a commercially available software (Stata 15/MP2).

## Results

### Clinical Presentation, Radiology, and Pathology

Ninety-two patients met the inclusion criteria. There were 53 girls (58%) and 39 boys (42%). The mean age at the time of diagnosis was 83 months (range, 1 month–17.3 years). Thirty-four tumors were low grade (WHO grade 1–2), and 58 tumors were high grade (WHO 3–4). Details of clinical and radiological presentation and tumor anatomical features are shown in **Table 1**.

**Table 1:** Preoperative clinical, radiological, and pathological assessment.

		Total No. (%)	Telovelar No. (%)	Transvermian No. (%)
**Age at diagnosis**	<6 months	7 (7)	6 (86)	1 (14)
	6 months–5 years	21 (23)	14 (67)	7 (33)
	>5 years	64 (70)	31 (48)	33 (52)
**Incidental diagnosis**		3 (3)	3	0
**Neurological assessment^1^**	ICH	81 (91)	43 (53)	38 (47)
	Cerebellar syndrome	34 (38)	15 (47)	18 (53)
	CN palsy	29 (33)	16 (55)	13 (45)
	Various^2^	36 (39)	19 (52)	17 (48)
**Anatomical pattern**	Fourth ventricle (pure)	23 (25)	14 (61)	9 (39)
	+ Mes./Aq.	4 (4)	4	0
	+ Verm./Cerebell.	48 (53)	16 (33)	32 (67)
	+ CPA	4 (4)	4	0
	+ BS dorsally exophytic	12 (13)	11 (92)	1 (8)
	+ Entire PCF	1 (1)	1	0
**Lateral extension**	No	39 (42)	18 (46)	21 (54)
	Unilateral	34 (37)	21 (62)	13 (38)
	Bilateral	19 (21)	12 (63)	7 (37)
**Caudal extension**	No	23 (25)	12 (52)	11 (48)
	Magendie	37 (40)	15 (42)	22 (58)
	+ Cisterna magna	20 (22)	15 (75)	5 (25)
	+ Cervical spinal canal	12(13)	9 (75)	3 (25)
**BS involvement**	No	52 (57)	25 (48)	27 (52)
	BS infiltration	28 (30)	15 (54)	13 (46)
	BS dorsally exophytic	12 (13)	11 (92)	1 (8)
**Hydrocephalus**	No	22 (24)	15 (68)	7 (32)
	Yes	70 (76)	36 (51)	34 (49)
**Tumor volume^3^**	<15 cm^3^	28 (33)	20 (71)	8 (29)
	15–26 cm^3^	30 (34)	15 (50)	15 (50)
	>26 cm^3^	28 (33)	14 (50)	14 (50)
**Histological subtypes^4^**	Medulloblastoma	37 (40)	16 (43)	21 (57)
	Pilocytic astrocytoma	28 (30)	13 (46)	15 (54)
	Ependymoma	7 (8)	4 (57)	3 (43)
	Anaplastic ependymoma	6 (7)	5 (83)	1 (17)
	ATRT	6 (7)	5 (83)	1 (17)
	Various	8 (8)	8 (8)	0
**Oncogenic syndromes**	NF-1	4 (4)	2 (50)	2 (50)
	Turcot syndrome	1 (1)	0	1
**Gene mutations**	ALC-RET	1 (1)	1	0
	AUTS2	1 (1)	0	1

CN, cranial nerve; Mes., mesencephalic; Aq., aqueductal; Ver., vermian; Cerebell, cerebellar; CPA, cerebellopontine angle; BS, brainstem; ICH, intracranial hypertension; PCF, posterior cranial fossa; CE, contrast enhancement; ATRT, atypical teratoid/rhabdoid tumor; NF-1, neurofibromatosis type 1; ALC-RET, ALC-RET gene mutation; AUTS2, AUTS2 gene mutation.

^1^Most patients presented with more than one sign/symptom.

^2^Various: torticollis, evolutive macrocrania, diffuse hypotonia, opisthotonic posturing, irritability.

^3^We used two tertiles to split volumetric data into three groups. N = 86 computable MRI sequences for volumetric analysis.

^4^According to the 2016 WHO Classification of Tumors of the Central Nervous System.

Brain MRI was performed in 89 patients (3 not performed due to emergency surgery). In six patients, tumor volume was not computable due to lack of the specific postop MRI volumetric sequences. In the remaining 86 patients, median tumor volume was 25.21 cm^3^ (range, 0.529–137.696 cm^3^). Tumor volumes were classified into three homogeneous groups: <15, 15–26, and >26 cm^3^, using the two tertiles.

The main differences in the choice of the approach were dictated by the anatomical pattern of the tumor: telovelar was chosen exclusively for tumors of the aqueduct bulging in the fourth ventricle and intraventricular extension of CPA tumors and almost exclusively for dorsally exophytic brainstem tumors. For pure intraventricular tumors or for tumors expanding into the cisterna magna or in the cervical canal, the telovelar approach was predominantly chosen. The only group where the choice of transvermian was predominant was the group with extensive vermian infiltration. Telovelar approach was also preferred when the tumor was <15 cm^3^ (**Table 1**). Telovelar approach was more frequently chosen for all ependymoma subtype and for AT-RT (**Table 1**).

### Surgery

Telovelar approach was performed in 51 cases and transvermian approach in 41 cases. Details of operative features are listed in **Table 2**. Twenty-eight children (30%) were re-operated for progressing and/or relapsing disease. Multiple surgical procedures were performed in nine patients (range, 2–4 procedures for each patient).

**Table 2:** Surgery and postoperative clinical/radiological assessment.

	Total No. (%)	Missing data	Telovelar No. (%)	Transvermian No. (%)
**C1 laminectomy**	80 (88)	n = 1	51 (64)	29 (36)
**C1 + C2 laminotomy**	3 (3)	n = 1	3	0
**Intraoperative changes of CVP**	13 (16)	n = 11	11 (85)	2 (15)
**Macroscopic evidence of BS involvement**	38 (43)	n = 1	26 (68)	12 (32)
**Macroscopic evidence of CV infiltration**	48 (53)	n = 1	15 (31)	33 (69)
**Transient neurological deficit ^1^**	48 (53)	n = 1	26 (54)	22 (46)
Cerebellar syndrome	25 (28)		16 (64)	9 (36)
Upper CN palsy	23 (26)		14 (61)	9 (39)
Pyramidal syndrome	14 (16)		8 (57)	6 (43)
Cerebellar mutism	10 (11)		4 (40)	6 (60)
Dysphagia	7 (8)		4 (57)	3 (43)
Dysphonia	3 (3)		3	0
**Permanent neurological deficit^1^**	25 (31)	n = 11^2^	16 (64)	9 (36)
CN palsy	16 (20)		10 (63)	6 (47)
Cerebellar syndrome	14 (18)		7 (50)	7 (50)
Pyramidal syndrome	9 (11)		5 (56)	4 (44)
Dysphagia	2 (3)		1 (50)	1 (50
**Mechanical ventilation**		n = 1		
<48 h	80 (88)		41 (51)	39 (49)
>48 h	11 (12)		8 (73)	3 (27)
**Medical morbidity**	19 (21)		14 (74)	5 (26)
**CSF-related complications**				
Pseudomeningocele	21 (23)		9 (43)	12 (57)
CSF leak	7 (8)		4 (57)	3 (43)
CSF infections <1 month	4 (4)		2 (50)	2 (50)
CSF infections >1 month	1 (1)		0	1 (50)
**Residual disease volume**		n = 2		
No residual disease	57 (63)		28 (49)	29 (51)
<1.5 cm^3^	23 (26)		15 (65)	8 (35)
>1.5 cm^3^	10 (11)		6 (60)	4 (40)
**EOR^3^**		n = 2		
Total	57 (63)		29 (51)	28 (49)
Subtotal (>90%)	21 (24)		13 (62)	8 (38)
Partial (<90%)	12 (13)		8 (67)	4 (43)
**Location of residual disease^3^**	33	n = 2		
Brainstem/floor of the fourth ventricle	18 (55)		12 (67)	6 (33)
Fastigium/CV	10 (30)		6 (60)	4 (40)
Various	8 (42)		4 (50)	4 (50)
**Radiological adverse events**	18 (20)			
Intraventricular blood clots	8 (9)		2 (25)	6 (75)
Pericerebellar fluid collections	6 (7)		2 (33)	4 (67)
Ischemia (PICA territory)	1 (1)		1	0
Epidural hematoma	1 (1)		0	1
Cerebellar swelling	1 (1)		1	0
**CSF-diversion procedures^4^**	65 (93)			
ETV	43 (66)		22 (51)	21 (49)
EVD	13 (20)		5 (38)	8 (62)
VPS	9 (14)		8(89)	1 (11)
**Postoperative ETV patency (<48 h)**	29 (74)	n = 4	14 (48)	15 (52)
**Postoperative ETV failure (<48 h)**	10 (26)	n = 4	6 (60)	4 (40)
**Postoperative hydrocephalus**				
Preop. CSF-diversion procedures	14 (22^4^)		9 (64)	5 (36)
No preop. CSF-diversion procedures	2 (40^4^)		0	2
No hydrocephalus at onset	7 (32^4^)		5 (71)	2 (29)
**Permanent VPS**	23 (25)		17 (74)	6 (26)

CVP, cardiovascular parameters; CT, computed tomography; BS, brainstem; CV, cerebellar vermis; CN, cranial nerve; CSF, cerebrospinal fluid; EOR, extension of resection; ETV, endoscopic third ventriculostomy; EVD, external ventricular drainage; VPS, ventriculo-peritoneal shunt; preop., preoperative.

^1^Most patients presented with more than one sign/symptom.

^2^n = 11 patients died before 1 year of follow-up.

^3^n = 3 cases of multiple location.

^4^n = 65 (93%) of n = 70 patients with hydrocephalus at onset were treated with CSF-diversion procedures; n = 5 (7%) patients with hydrocephalus were referred directly to surgery; in n = 22 cases, preoperative hydrocephalus was not detected (see **Table 1**).

Early postoperative MRI studies showed complete removal in 57 cases (62%) and measurable residual tumor in 35 cases (38%). The volume of residual disease could be measured in 33 cases (missing MRI sequences in two cases). The average tumor residual volume was 1.316 cm^3^ (range, 0.016–4.231 cm^3^; median value, 0.9875 cm^3^). Large residuals (>1.5 cm^3^) were equally distributed between telovelar (6) and transvermian approaches (4), whereas smaller residuals (<1.5 cm^3^) where more frequently left using the telovelar (15 cases) than the transvermian approach (8 cases), but these differences were not significant. Details of extension of resection (EOR%), volumetric quantification of residual disease (cm^3^), and specific locations of residual disease are listed in **Table 2**.

### Surgery-Related Complications

Postoperative radiological adverse events were identified on 18 postoperative MRI (20%) (**Table 2**), and only 2 of these events required surgical treatment: within 48 h in one case (cerebellar swelling, treated with EVD) and beyond 48 h in another one case (epidural hematoma from Mayfield pin).

Postoperative CSF-related complications are described in **Table 2**. Pseudomeningocele was resolved by lumbar puncture in almost all cases (mean number, n = 2; range, 1–4) except in two patients who required lumbar spinal drainage.

### Management of Hydrocephalus

Hydrocephalus at onset required CSF-diversion procedures in 65 cases (93%). Early radiological signs of ETV failure were detected in 10 cases (26%). Among treated cases, 14 children (22%) developed postoperative hydrocephalus.

Seven cases of mild preoperative hydrocephalus were referred directly to surgery within 24 h and developed early postoperative hydrocephalus in two cases. Seven patients without preoperative hydrocephalus required CSF-diversion procedures.

Overall, in the long term, 23 children of our population required permanent VP shunt (25%). Patients treated preoperatively by ETV required permanent VP shunt in 19% of the cases, whereas patients treated with pre- or intraoperative EVD required permanent VP shunt in 8% of the cases. Other differences in hydrocephalus treatment and outcome between the two groups are shown in **Table 2**.

### Postoperative Course

According to the aforementioned diagnostic criteria, cerebellar mutism was identified in 10 children (11%) (**Table 2**). In all cases, the onset of speech loss appeared within 4 days after surgery. The duration of mute phase lasted up to 15 days in eight cases (80%) and up to 30 days in two cases (20%); nine patients (90%) exhibited dysarthria after remission of mutism, with long-term persistence of motor speech deficits in six cases (60%).

When considering the approach chosen, no significant difference in the onset of CM was detected between telovelar and transvermian approach (**Table 3**).

**Table 3:** Surgical approach and postoperative outcomes.

	Transient deficit											
	Yes		No				Univariate analysis			Multivariate analysis*		
	n	%	n		%		OR	95% CI	p	OR	95% CI	p
*Telovelar*	26	52	24		48							
*Transvermian*	22	54	19		45		1.07	0.47–2.44	0.88	1.28	0.39–4.15	0.68
	**Permanent deficits**											
	*Yes*		*No*									
*Telovelar*	16	36	28		64							
*Transvermian*	9	24	28		76		0.56	0.21–1.48	0.25	0.86	0.21–3.43	0.83
	**Ventilation**											
	*>48 h*		*<48 h*									
*Telovelar*	8	16	41		84							
*Transvermian*	3	7	39		93		0.39	0.10–1.59	0.19	1.26	0.17–9.61	0.82
	**Cerebellar mutism**											
	*Yes*		*No*									
*Telovelar*	4	8	46		92							
*Transvermian*	6	15	35		85		1.97	0.52–7.51	0.32	0.81	0.16–4.12	0.81
	**Medical events**											
	*Yes*		*No*									
*Telovelar*	14	28	36		72							
*Transvermian*	5	12	37		88		0.35	0.11–1.06	0.06	0.26	0.06–1.23	0.09
	**Residual disease**											
	***No***		***<1.5 cm^3^***		***>1.5 cm^3^***		Univariate analysis**			Multivariate analysis**		
	***n***	***%***	***n***	***%***	***n***	***%***	***OR***	***95% CI***	***p***	***OR***	***95% CI***	***p***
*Telovelar*	28	57	15	31	6	12						
*Transvermian*	29	71	8	19	4	10	0,58	0.24–1.37	0.21	0.44	0.12–1.60	0.22

^*^Penalized maximum likelihood estimation.

^**^Ordered logistic model.

Postoperative new-onset transient neurological deficits were assessed in 48 cases (53% of 91 cases analyzed; multiple deficits in 69%) and are listed in **Table 2**. No differences were found between the two approaches (**Table 3**).

Postoperative permanent neurological deficits (persistent >1 year of follow-up) were assessed in 25 patients (31% of 81 patients: 11 patients died before 1 year of follow-up; multiple permanent deficits in 56%) (see **Table 2**). This incidence was not modified by the choice of the approach (**Table 3**).

Postoperative new onset medical events and duration of mechanical ventilation following the first surgery are shown in **Table 2**, and both were not influenced by the surgical approach (**Table 3**).

Average intensive care unit length of stay, consequent to the first surgery, was 3 days (range, 1–26 days), and no differences were found between the two approaches (**Table 3**).

Residual disease was more often detected on immediate postop MRI after telovelar approach, but the difference was not significant (**Table 3**).

Overall, in univariate and multivariate analyses, neither telovelar nor transvermian approach modified significantly any postoperative outcome analyzed.

### Follow-Up and Adjuvant Therapy

Mean clinical–radiological follow-up was 55.5 months (range, 0–136 months). In detail, during radiological surveillance after first radical surgery (n = 57 cases of no residual disease), 43 cases (75%) remained stable without relapse, and 14 cases relapsed (25%). Among the cases with residual disease (n = 35), 18 cases (51%) did not progress, 14 cases (40%) progressed, and 3 cases (9%) progressed and, then, after surgical resection, relapsed. Twenty-one cases (23%) suffered from tumoral dissemination or metastasis.

According to the last clinical examination and radiological evaluation, we categorized our patients in three main groups: ANED (alive with no evidence of disease), n = 45 cases (49%); AWED (alive with evidence of disease), n = 24 cases (26%); and DOD (died of disease), n = 23 cases (25%).

Two patients died due to chemotherapy complications. One patient, suffering from tumor predisposition syndrome, died due to leukemia.

No intraoperative death was documented. Eleven children died before 1 year from the diagnosis; of these, 1 perioperatory death was assessed (a child admitted in coma and bilateral mydriasis), 1 patient died of postoperative respiratory complications, and 9 deaths were related to the aggressive tumor behavior.

**Figure 2:**
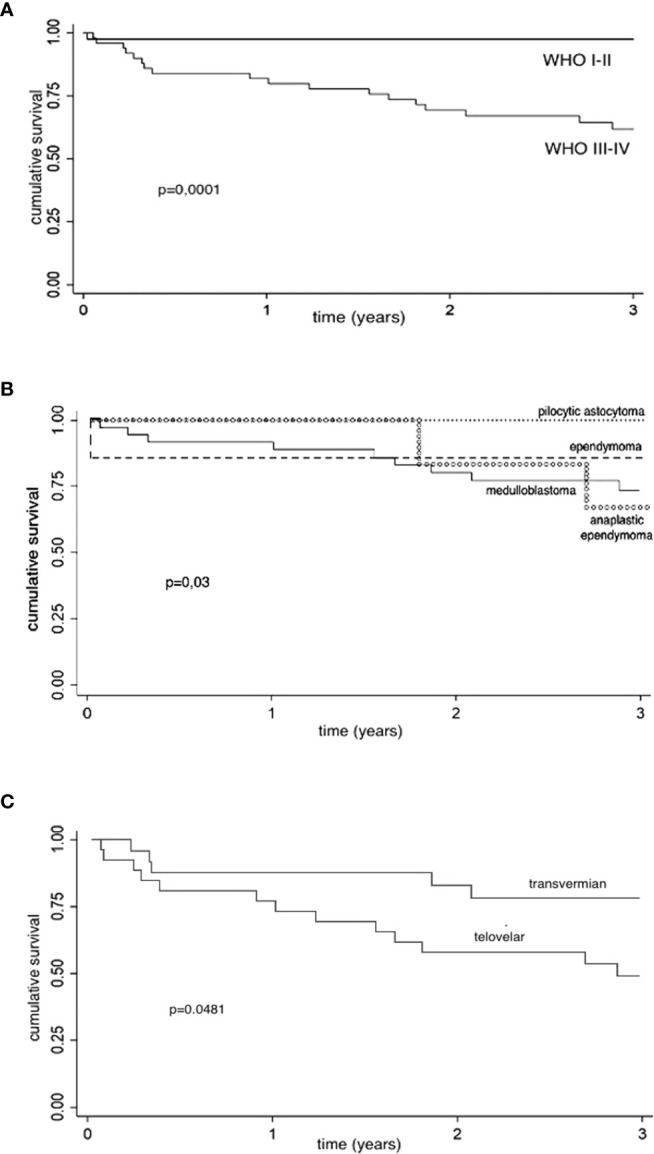
Kaplan–Meier survival estimates. CS, cumulative survival; CI, confidence interval; y, year. **(A)** Kaplan–Meier survival estimates (histological grading): 1- and 3-year CS are analyzed in relation to histological grading (WHO 1–2 vs. WHO 3–4), showing a better 1- and 3-year CS for low-grade group (log-rank test, p = 0.0001). Low-grade tumors: 1-year CS, 97.6% (95% CI: 83.9%–99.7%); 3-year CS, 97.6% (95% CI: 83.9%–99.7%). High-grade tumors: 1-year CS, 82% (95% CI: 68.2%–90.2%); 3-year CS: 62% (95% CI: 46.4%–74.2%). **(B)** Kaplan-Meier survival estimates (histology): 1- and 3-year CS are analyzed in relation to histology (log-rank test, p = 0.03). Pilocytic astrocytoma: 1- and 3-year CS, 100%. Non-anaplastic ependymomas: 1-y CS, 85.7% (95% CI: 33.4%–97.9%); 3-year CS, 85.7% (95% CI, 33.4%–97.9%). Medulloblastomas: 1-year CS, 91.9% (95% CI, 76.9%–97.3%); 3-year CS, 73.6% (95% CI: 55.1%–85.4%). Anaplastic ependymomas: 1-year CS, 100%; 3-year CS, 66.7% (95% CI: 19.5%–90.4%). **(C)** Kaplan–Meier survival estimates (surgical approach for high-grade tumors): Considering the choice of surgical approach for high-grade tumors (WHO 3–4), we found better 1- and 3-years CS of transvermian approach when compared to telovelar approach with statistical significance (log-rank test, p = 0.048). Telovelar approach: 1-year CS, 76.9% (95% CI: 55.7%–88,9%); 3-year CS, 48.8% (95% CI, 28.5%–66.4%). Transvermian approach: 1-year CS, 87.5% (95% CI: 66.1%–95.8%); 3-year CS, 78% (95% CI, 54.8%–90.3%). The power of predicting factors (surgical approach for high-grade tumors and residual tumor volume) is evaluated in a Cox proportional hazard model (**Table 4**).

One- and 3-year CS were analyzed in relation to histological grading (WHO 1–2 vs. WHO 3–4), showing, as expected, a better 1- and 3-year CS for low-grade group (log-rank test, p = 0.0001) (see **Figure 2A**).

One- and 3-year CS for medulloblastoma, pilocytic astrocytoma, ependymomas, and anaplastic ependymomas are reported in **Figure 2B** (log-rank test, p = 0.03).

Considering the choice of surgical approach for high-grade tumors (58 cases), we found a better 1- and 3-year CS of tumors operated through a transvermian approach when compared with tumors operated through the telovelar with statistical significance (log-rank test, p = 0.0481) (**Figure 2C**). The power of predicting factors (surgical approach and postoperative residual tumor volume), evaluated in a Cox proportional hazard model, show that higher scores on postoperative residual tumor volume are associated with poorer prognosis (HR, 2.53). This is statistically significant (p = 0.003) (**Table 4**).

**Table 4:** Cox regression (High-grade tumors).

	*HR*	*z*	*p>ǀzǀ*	*95% CI*
Surgery	0.47	−1.39	0.165	0.16–1.37
Residual volume (cm^3^)	2.53	2.99	0.003	1.38–4.64

HR, hazard ratio; CI, confidence interval; p, p-value.

## Discussion

The goals of fourth ventricular tumors surgery are to 1) obtain the largest possible safe tumor resection, 2) restore CSF circulation releasing the fourth ventricle outlets, 3) decompress the brainstem, and 4) obtain tissue sample for pathological and molecular analysis (4). Historically, the oldest and the most intuitive route to the fourth ventricle was to remove part of a cerebellar hemisphere (1). Dandy stated that the inferior cerebellar vermis could be incised at its center and split on the cerebellar suboccipital surface without any significant functional consequence, taking care to avoid excessive surgical manipulation and damage to the dentate nuclei (3).

Since Dandy’s original report (3), the transvermian route has been the most used approach to access the fourth ventricle (13). Dailey (12) and other authors (15–24) considered the vermian incision by default as the main responsible for cerebellar mutism, although clear evidence of this is still lacking. Moreover, the reported more limited control of the laterality offered by this approach motivated some authors (5–11) to explore the natural corridor of the cerebellomedullary fissure (CMF) opening of the tela choroidea and the inferior medullary velum, to avoid any incision of cerebellar parenchyma. Matsushima described three main ways to dissect the CMF, approaching it on its medial or lateral side, or both (5–11).

The medial variant of the CMF opening is the so-called “telovelar approach” and was developed for intra-CMF and/or intraventricular lesions extending also into the cerebellomedullary cistern (CMC), approached *via* the midline suboccipital route (5, 6, 8, 9, 11). In cadaveric specimens, the telovelar approach allows a better operative control of the lateral aspects of the fourth ventricle (25, 26), and C1 laminectomy nullifies the advantage of the transvermian approach in terms of operative working angle when accessing the rostral fourth ventricle (26). These findings are coherent with our surgical experience.

The choice of surgical route (telovelar vs. transvermian) may depend on thorough evaluation of preoperative imaging, intraoperative features, and surgeon’s preference/experience. The trend of recent literature considers the telovelar approach protective towards cerebellar mutism attributed to the vermian incision and splitting (20–24), leading us to investigate this topic in our cohort.

Postoperative CM [reported incidence, 8–32% (14, 27, 28)] is characterized by delayed onset mutism (24–48 h after surgery), reduced speech, limited duration and spontaneous recovery, usually associated with other deficits of cognitive, affective, and motor functions (13, 14, 29, 30).

Immediately after the transient mute phase, almost all children experience dysarthria (30, 31), as we have also seen in our patients (90% of 10 CM cases, in our cohort), with long-term persistence of motor speech deficits in 60% of our cases, according to other reports (30).

There are two main types of motor speech deficits: 1) a pure dysarthria with normal cognitive functions and 2) an apraxic language disorder with more complex neuropsychological correlates (30).

The severity of associated neuropsychological deficits after surgery was found to be a negative prognostic factor for long-term motor speech deficits, in terms of clinical/neuropsychological impact (30). Full recovery is often incomplete in cases of apraxic dysarthria (30, 31).

However, there is no substantial consensus regarding other prognostic factors, such as the age of onset, for long-term sequelae of CM (30).

Many surgical series, focused exclusively on the telovelar approach, show the beneficial result of this approach in preventing or reducing CM (15–24, 32). When the telovelar approach is compared with the transvermian approach (33, 34), it seems to be protective for postoperative CM and neurological morbidity, although statistical significance was only reported by Ferguson et al. (33) in a mixed adult/child series. The work of Ebrahim et al. (34) does not include adequate statistical analysis.

However, when considering larger series in everyday clinical practice, the reported advantages of telovelar approach are less evident.

Cobourn et al. (35) reported that in pediatric medulloblastomas, vermian incision seems to be a risk factor for CM.

In a retrospective multicenter analysis of 263 pediatric patients harboring posterior fossa tumors, Renne et al. (36) reported no statistical correlation between the surgical approach and postoperative CM.

Recently, Toescu et al. (37) retrospectively analyzed 167 fourth ventricle tumors in a case series of only pediatric patients, showing no significant difference in the rate of CM between telovelar or transvermian approach and no statistically significant relationships between cerebellar mutism syndrome and surgical approach.

Among the few purely pediatric fourth ventricular tumor case series published in the literature (**Table 5**), a retrospective head-to-head comparison between telovelar and transvermian approach was carried out only in one case (Toescu et al. (37). In Cobourn et al. (35), the frequency of telovelar approach was not reported. In all other studies, the impact of the surgical approach on CM has been retrospectively analyzed only in children treated *via* the telovelar route, showing a wide variability in incidence of mutism (0–30%) (**Table 5**).

**Table 5:** Surgery of fourth ventricle tumors: published pediatric case series.

Authors, year	No of patients	Telovelar approach (%)	Transvermian approach (%)	CMS (%)	GTR (%)
Kellogg & Piatt, 1997 (15)	11	100	0	0	81.2
Rajesh et al., 2007 (18)	15	100	0	13.3	93.3
Zaheer& Wood, 2010 (19)	20	100	0	30	70
Qiu et al., 2016 (22)	26	100	0	7.7	84.6
Eissa, 2018 (24)	40	100	0	2.5	45
Atallah et al., 2019 (32)	44	100	0	13.6	84.1
Cobourn et al., 2020 (35)	63	*	53.8	10.8	NR
Toescu et al., 2020 (37)	167	33.0	64.1	28.7	70.7
*current series*	*92*	*55*	*45*	*11*	*62*

CMS, cerebellar mutism syndrome; GTR, gross total removal; NR, not reported.

*Frequency of telovelar approach: not reported.

When the telovelar approach fails to reduce the incidence of CM, other explanations are proposed (30, 38).

In our series, the two approaches were not randomized and were not considered as alternative but complementary, with specific indications for each approach only based on careful and thorough evaluation of preoperative imaging.

Transvermian approach was chosen for midline fourth ventricular tumors associated with extensive lower vermian infiltration and/or rostral extension up to the fastigium and upper vermis infiltration. The telovelar approach was chosen for tumors filling only the fourth ventricle and/or bulging in the cisterna magna through an enlarged Magendie, with little or no vermian infiltration, lateral extension, and CPA extension (**Table 1**). Using these criteria, the overall incidence of CM remained in the lower range (n = 10 cases; 11%) of those reported in the literature (27, 28, 36–38) regardless of the approach used (**Table 5**).

According to our data, the development of CM after posterior fossa surgery in children appears to be a more complex phenomenon, requiring a combination of 1) surgery-unrelated factors (e.g., location of the tumor, medulloblastoma histology, brainstem infiltration) and 2) surgery-related factors (e.g., surgical manipulation near the dentate nuclei or the superior/middle cerebellar peduncles causing injury to the dento-rubro-thalamo-cortical pathways, inadequate use of self-retaining retractors and ultrasonic aspiration) (28, 35, 36, 38, 39). The risk factors identified in most papers (28) are mainly surgery unrelated: midline location (vermis and/or fourth ventricle), brainstem infiltration/compression, medulloblastoma histology with higher risk for 3–4 molecular subgroups (40), and tumor size (41, 42).

The choice of surgical approach did not significantly modify any considered postoperative outcome (**Table 3**), in contrast with that of Ferguson et al. (33) and according to the recent findings of Toescu et al. (37).

Our data show transient neurological deficits in 53% of children and permanent neurological morbidity in 31% of cases (**Table 2**) with prevalence of motor/cerebellar deficits and cranial nerve impairment. In a mixed adult/child retrospective series of 55 surgically treated fourth ventricular tumors, Ferguson et al. (33) describe neurological complications in 76% of cases. Toescu et al. (37) report postoperative morbidity in 70.7% of 167 children with fourth ventricular tumors. Despite the differences in data collection and classification, our findings are similar to those presented in previous series.

Radiological adverse events (20% of cases in our cohort, see **Table 2**) required neurosurgical treatment only in two cases (see *Results*). The low incidence of postoperative neurosurgical complications requiring reoperation is in line with Toescu et al. (37).

It could be hypothesized that telovelar approach could offer less good visibility or less comfortable working angles in the area of the fastigium and fourth ventricle roof, but in fact, the measurable postoperative residual tumor was left adherent to the brainstem or in the fastigium area indifferently from the approach used.

The senior surgeon operating and/or closely supervising all the surgeries trained in the 1990s in the transvermian era but started to use progressively the telovelar in the early 2000s after the publication of Mussi (7), so he could be considered skilled enough also in the telovelar approach at the early time of recruitment of this study, canceling the possible bias of different learning curve.

The finding that high-grade tumors operated on through the transvermian approach have a better CS than those operated on through the telovelar approach should only be considered as a confirmation that, in our series, the choice of the surgical approach was highly dependent on anatomical presentation and the typology of tumor and the telovelar being mainly chosen in two aggressive histologies (AT-RT, anaplastic ependymoma). Moreover, the significant impact of the choice of surgical approach (**Figure 2C**) should be reconsidered when the factor of postoperative residual tumor volume is taken into account (**Table 4**).

Therefore, in this unicentric, single-surgeon retrospective series, when surgical approach was chosen only on the basis of a rational and thorough examination of preoperative images, cerebellar mutism remained approximately 11% whatever the approach used. In our hands, none of the two surgical approaches proved to be superior to the other in terms of quality of resection and postoperative complications. For these reasons and due to the inconsistency of the current literature relative to this subject, we will continue to choose the approach on the basis of the preoperative MRI anatomical features and intraoperative characteristics until further evidence will become available. Telovelar approach will be preferred whenever anatomically suitable for its higher anatomical respect of cerebellar parenchyma, but transvermian approach will continue to be part of our surgical armamentarium in cases where extensive vermian infiltration or unusual dorsal extension will lead us to consider the latter approach safer for the patient.

## Conclusion

In our experience, the choice of the surgical approach (telovelar vs. transvermian) to fourth ventricular tumors in children did not significantly modify any considered postoperative outcome, including the incidence of postoperative CM.

Our findings offer significant data to reconsider the real impact of the choice of surgical route to the fourth ventricle on the incidence of CM and surgery-related morbidity. Surgical approach to the fourth ventricle, like all surgical approaches, should be individualized according to the location of the tumor, degree of vermian infiltration, and lateral and upward extension. Surgeons should fully master both approaches and choose the one that they consider the best for the patient according preoperative imaging evaluation.

## Data Availability Statement

The raw data supporting the conclusions of this article will be made available by the authors, without undue reservation.

## Ethics Statement

The present study was approved by the Cardarelli-Santobono Hospitals Ethics Committee (Cod.Reg. RGP2020 - Prot.N. 00022209 - 21/09/2020).Written informed consent from the participants’ legal guardian/next of kin was not required to participate in this study in accordance with the national legislation and the institutional requirements.

## Author Contributions

NO and GC: study design and manuscript conception. NO: data collection. NO and GC: data revision and interpretation and manuscript drafting and revision. FS, CC, and NO: statistical analysis. CR, GC, and NO: imaging data interpretation. NO, VO, and CR: volumetric analysis. LDM, LQ, MdS, and NO: oncological follow-up data analysis. NO, GC, PS, CRu, and GM: surgical videos and operative reports interpretation. All authors contributed to the article and approved the submitted version.

## Funding

Funding was provided by Public Charity Fund of our Hospital, which is the “Fondazione Santobono-Pausilipon,” http://www.fondazionesantobonopausilipon.it.

## Conflict of Interest

The authors declare that the research was conducted in the absence of any commercial or financial relationships that could be construed as a potential conflict of interest.

## Publisher’s Note

All claims expressed in this article are solely those of the authors and do not necessarily represent those of their affiliated organizations, or those of the publisher, the editors and the reviewers. Any product that may be evaluated in this article, or claim that may be made by its manufacturer, is not guaranteed or endorsed by the publisher.
